# Inter-Ply Slipping Behaviors and Kinetic Equation of Carbon Fiber-Reinforced Epoxy Prepregs for Hot Diaphragm Preforming

**DOI:** 10.3390/ma17225592

**Published:** 2024-11-15

**Authors:** Haoxuan Zhang, Jintong Liu, Congfa Zhang, Hongfu Li, Guangquan Yue, Baozhong Sun, Boyan He

**Affiliations:** 1 Shanghai Collaborative Innovation Center of High Performance Fibers Composites (Province-Ministry Joint), Center for Civil Aviation Composites, Donghua University, Shanghai 201620, China; 2 Shanghai Frontiers Science Center of Advanced Textiles, College of Textiles, Donghua University, Shanghai 201620, China; 3 Beijing Institute of Spacecraft System Engineering, Beijing 100094, China; 4 School of Materials Science, Engineering University of Science and Technology Beijing, Beijing 100083, China; 5 Faculty of Engineering, The Hong Kong Polytechnic University, Hong Kong 999077, China

**Keywords:** carbon fiber, inter-ply slipping, wrinkles, hot diaphragm preforming

## Abstract

Wrinkles are urgent problems to be solved in the process of hot diaphragm preforming. Inter-ply slipping resistance is one of the causes of wrinkles. In this paper, based on the vertical inter-ply slipping test system, the inter-ply slipping behaviors of carbon fiber-reinforced epoxy resin composite prepregs were characterized. The mechanism of wrinkles caused by inter-ply slipping resistance was analyzed. According to the different characteristics expressed by the fiber and resin during the slip process, the inter-ply slipping behaviors of the prepregs were divided into three stages. The effect of temperature on the inter-ply slipping stresses was shown. The temperature will affect the viscosity of the prepregs. When the viscosity of the prepregs is low, the inter-ply slipping resistance will decrease. Based on the Coulomb friction law and the hydrodynamic equation, the inter-ply slipping kinetic equation of the prepregs was established. The inter-ply slipping kinetic equation was introduced into the ABAQUS main program by the ‘vfriction’ subroutine. The introduction of inter-ply slipping dynamics improved the accuracy of predicting the shape and position of wrinkles.

## 1. Introduction

Carbon fiber-reinforced resin matrix composites have been widely used in aerospace [1]. The uncertainty surrounding the manufacturing process of composite components has brought many challenges to the application of composite materials [2]. Hot diaphragm preforming technology is based on the process technology commonly used in the manufacture of large-scale composite preforms under the automatic laying process of composite materials. For the preforming of some structural parts, hot diaphragm preforming technology can quickly complete the preforming process and save time for subsequent curing and other processes. The composite components usually have many defects due to process parameters, the molding environment and other factors [3,4,5]. In general, the defects comprise warpage, deformation, delamination, wrinkles and other issues. Controlling the forming quality of composite preforms is an effective means to ensure the quality of composite components [6,7,8].

Wrinkles [9] are a common defect in preforms during the process of hot diaphragm preforming. In the forming process of the preform, due to the deformation of the shape of the component, the pressure leads to a change in the curvature of the component, the laying draft force and other factors [10,11,12]. The relative slip effect will occur between the prepreg layers. The inter-ply slipping behaviors of prepreg are usually incomplete [13]. The influence of inter-ply slipping resistance leads to this situation. This incomplete slip behavior leads to the generation of wrinkles, especially in parts with large curvature changes, such as R angles. It is beneficial to control the generation of wrinkles to ensure the accurate characterization of inter-ply slipping behaviors and slip resistance of prepregs [14,15,16].

The measured inter-ply slipping behaviors and slip resistance of prepregs can be verified by finite element tools [17]. ABAQUS software (ABAQUS/CAE 2023) with a subroutine interface can be used to characterize the inter-ply slipping characteristics of prepregs. Many scholars use subroutine tools to apply the actual characteristics of materials to finite element work, such as unevenly forming temperature fields, material constitutive relationships, and so on. The corresponding subroutine can be compiled according to the measured results of inter-ply slipping of prepregs [18,19,20]. The inter-ply slipping behaviors of the prepreg is input into the main program [21]. With the help of subroutine tools, the inter-ply slipping behaviors of prepregs are expressed in finite element simulations, so that the inter-ply slipping behaviors of prepregs can be applied to finite element simulations.

In this paper, the inter-ply slipping behaviors of carbon fiber-reinforced unidirectional composite prepregs were characterized. The effect of temperature on the inter-ply slipping behaviors of prepregs was investigated. The slip behavior between prepregs was analyzed by Coulomb’s law and fluid dynamics modeling. The inter-ply slipping behaviors of prepregs were inserted into ABAQUS finite element simulation by using the ‘vfriction’ subroutine. The ‘vertical‘ friction test was simulated by ABAQUS software to verify the correctness and effectiveness of the subroutine’s compilation results.

## 2. The Causes of Wrinkles

**Figure 1:**
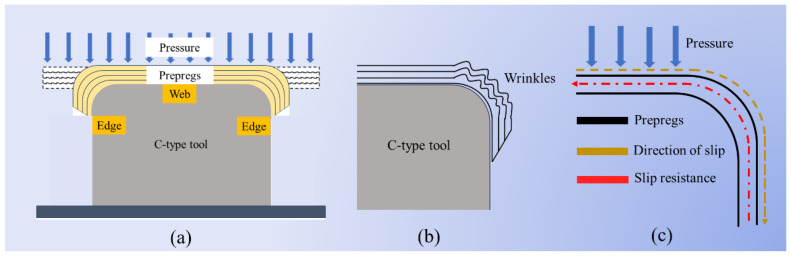
Process of hot diaphragm and generation of wrinkles; (**a**) Prepreg bonding tooling. (**b**) The shape of the wrinkles; (**c**) The cause of wrinkles.

During hot diaphragm preforming, due to the factors of pressure and gravity, the prepreg layer begins to bend and fit the tooling in its flat state, and finally forms (Figure 1a). C-type tooling was taken as an example. When the prepregs were bent, the shape of the tooling limited the bonding sequence of the prepregs, and the prepregs at the web position always contacted the mold first. Under the action of pressure, the bonding of the prepregs gradually extends from the web to the side edge. At the R corner, due to the thickness of the prepregs, the bending path of the outer prepreg is always longer than that of the inner prepreg. This path difference leads to the relative slip between the layers of the prepreg (Figure 1c). At the interlayer interface of the prepreg, contact resistance is generated due to the stagnation of the resin and the contact friction of the fiber. This resistance will hinder the slip of the prepreg, resulting in wrinkles (Figure 1b). This contact resistance is called inter-ply slipping resistance, and the change in resin and fiber during the slip process is called the inter-ply slipping behavior of prepregs. The inter-ply slipping resistance is distributed on the entire prepreg contact interface. The wrinkles have the possibility of appearing at any position of the tooling.

## 3. Materials and Methods

### 3.1. Material

The experimental material is continuous carbon fiber reinforced resin matrix composite prepreg (AVIC Composites Co., Ltd., Nantong, China). The fiber is T300 grade carbon fiber. The matrix is epoxy resin. The mass fraction of carbon fiber is 35%. The shape of the fiber is a cylinder, and the cross-sectional shape of the fiber is a circle. The prepreg should be placed at room temperature for 12 h before use, and it can be used after it is completely thawed. Test specimens were cut using a cutting machine (DCS-2506-24, purchased from Gerber Company, Florham Park, NJ, USA). The angle of the fiber in the prepreg is 0°.

### 3.2. Inter-Ply Slipping Test

**Figure 2:**
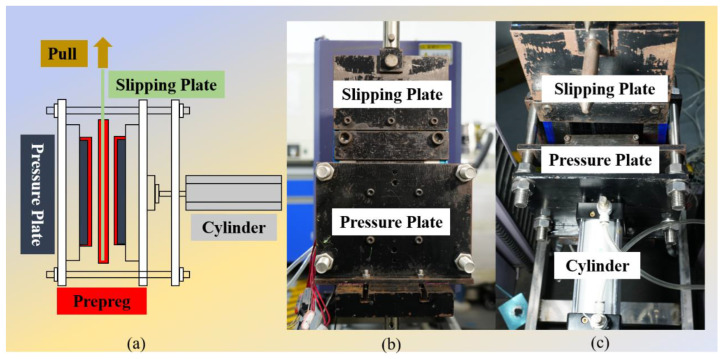
Schematic diagram of inter-ply slipping test device: (**a**) structural diagram; (**b**,**c**) device physical diagram.

The Figure 2 shows the structure of the inter-ply slipping test device. The size of the prepregs on the pressure plates is 190 mm × 100 mm, and the size of the prepreg on the slipping plate is 460 mm × 100 mm. The angle of the fiber in the prepreg pasted on the slipping plate and the pressure plate (Figure 2b) is 0°. The length direction of the test spline is the 0° direction of the prepreg. The complete testing process is being explained. Firstly, the prepregs with a size of 190 mm × 100 mm was attached to the pressure plates, and the size of 460 mm × 100 mm was attached to the slip plate. The edge of the prepreg is fixed using a press strip. The prepregs were difficult to dissociate during the test process. The contact area between prepregs is 100 mm × 100 mm, and the slip area is also 100 mm × 100 mm. Next, the whole test system is installed on the tensile machine (WANCE, ETM204C, Hubei Wance Testing Machine Co., Ltd., Wuhan, China). The heating will be opened, if the inter-ply slipping behaviors of the prepreg at a certain temperature needs to be measured. After the prepreg temperature rises to the test temperature, it is kept for 15 min to ensure that the prepreg temperature is uniform. Finally, the tensile machine was started and the test begun. The sliding plate was pulled by the tensile machine and begun to slid. The slip velocity is 1 mm/min. When the slip distance reaches 10 mm, the test ended and the curve was recorded. Repeat five tests and record the data. The cylinder pressure has been 0.1 MPa during the test. The similar products of the system have also been applied by Zhao [22] and Wang [23].

## 4. Mechanical Model

**Figure 3:**
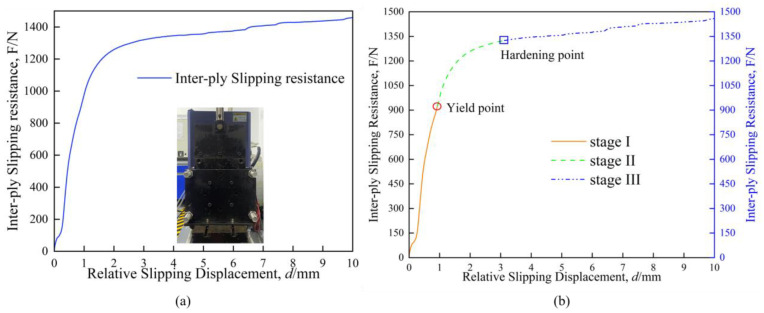
(**a**) the curve of inter-ply slipping resistance test; (**b**) Three-stage analysis of inter-ply slipping resistance curve.

The Figure 3a shows the inter-ply slipping resistance test curve tested by the vertical inter-ply slipping test system. The change of fiber and resin behaviors lead to the complexity of the change of inter-ply slipping resistance. According to the characteristics of resin and fiber expressed in interfacial friction, the inter-ply slipping behaviors of prepreg is divided into three stages. In the stage I, the contact interface between the prepreg layer and the layer is filled with resin. The inter-ply slipping at stage I mainly occurs in the flow of resin. It can be expressed by fluid dynamics equation [24]. The fluid dynamics model is shown in Formula (1).

(1) $$\tau =\frac{\eta }{h}v$$

where the *τ* is the slip stress. The *η* is the viscosity of fluid. The *h* is the height of the fluid layer. The *v* is the slip velocity of a contact surface relative to another contact surface.

The inter-ply slipping enters the stage II, as the slip proceeds. In the second stage, the height of the resin layer gradually decreases, and the softening flow effect of resin increases gradually. Therefore, the starting point of the second stage is called the ‘yield point’. The pressure causes the resin to penetrate between the fibers. The thickness of the resin layer at the contact interface decreases. Fiber bare leakage can enter the contact interface. The slipping behaviors between the fiber surfaces is involved in the inter-ply slipping behaviors of the prepregs. The slipping behaviors of the fiber surface can be expressed by the Coulomb friction law. The Coulomb friction model is shown in the Formula (2).

(2) f=μN

where *f* is frictional force. The *μ* refers to the dynamic friction coefficient. The *N* is the normal pressure of the friction interface. In the stage II, both the effect of resin flow and the friction effect of fiber surface are involved.

The Coulomb friction model is suitable for the contact surface is solid. When the contact surface is completely filled with fluid, the fluid dynamics model is available. The contact interface between the prepreg layers of the composite material has both fibers and resins. The contact interface of composite prepreg is a semi-solid and semi-fluid interface at stage II. The slip mechanical characteristics of the prepreg contact interface cannot be accurately expressed by adapting to the Coulomb model alone or using the hydrodynamic model alone. The inter-ply slipping friction model established by comprehensively considered the Coulomb friction and fluid dynamics [25]. The model is shown in Formula (3).

(3) $$\tau =\mu \cdot P_{N}+\eta \cdot \gamma$$

where the $P_{N}$ is the normal pressure of the friction interface. The γ is relative slip velocity of contact surfaces.

In the stage III, the resin basically penetrates between the fibers, and the friction effect on the fiber surface plays a leading role. In the stage III, the resin flow effect basically disappears, the fiber contacts directly, and the contact medium becomes hard. Therefore, the starting point of stage III is called ‘hardening point’. The Coulomb friction law is used to express the inter-ply slipping behaviors of the stage III.

## 5. Results and Discussions

### 5.1. Test Results at Different Temperatures

**Figure 4:**
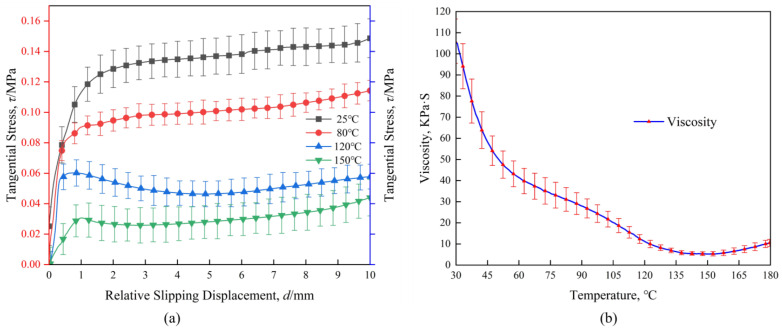
(**a**) Inter-ply slipping test results at different temperatures; (**b**) Viscosity-temperature curve of prepregs.

Figure 4a shows the different inter-ply slipping behaviors of prepregs at different temperatures. The inter-ply slipping behaviors of prepreg at different temperatures shows a classic three-stage distribution. In the stage I, the slipping stress is mainly caused by the fluidity of the resin and the gradual stabilization of the tensile rate. The stabilization process of the tensile rate of the testing machine is basically unchanged. Therefore, the slope of the slipping stress curve at the stage I of the prepreg at 4 temperatures is roughly the same. The slope of the 150 °C curve at the stage I is small, which was considered to be caused by the low viscosity leading to the premature entry of the slipping behaviors into the stage II. The difference in the maximum slip stress at the stage I at each temperature was due to the difference in the viscosity of the resin (Figure 4b). The stage II of slipping behavior is due to the gradual infiltration of the resin into the gap between the fibers, the flow effect of the resin is gradually reduced, and the fiber surface is gradually connected to the slipping interface, resulting in the slipping behaviors from the flow of the resin to the contact friction of the fiber surface. Due to the decrease of resin viscosity, the fluidity of the resin is enhanced, and it is easier to penetrate into the gap between the fibers, and the fibers enter the slipping interface faster. The range of the stage II also decreased with the decreased of resin viscosity. The inter-ply slipping behaviors enters the stage III faster. The stage III of the slipping behaviors is mainly caused by the mutual contact friction on the surface of the fiber. At this time, most of the resin has infiltrated between the fibers. However, the resin is not considered to be completely squeezed out of the slipping interface, and the role of the residual resin at the slipping interface is similar to ‘ lubricant ‘ and will exist for a long time, which is different from previous work [23]. The lower the viscosity of the resin, the better the fluidity and the better the lubrication effect. In the stage III, although the friction behaviors of the fiber surface leads to the slipping stress, the difference of the slipping stress value was caused by the different lubrication effect of the resin.

In summary, the difference of inter-ply slipping behaviors of prepregs at different temperatures is mainly due to the difference of resin viscosity, which affects the fluidity of resin and leads to the difference of inter-ply slipping stress value. However, the three stages of inter-ply slipping behaviors of prepregs are suitable for each temperature.

### 5.2. Slip Dynamic Model

**Figure 5:**
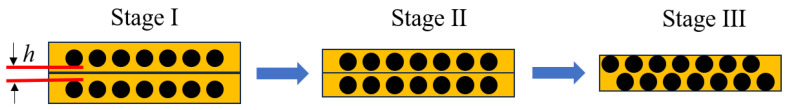
The change of the behaviors of resin and fiber in the process of inter-ply slipping test.

The changes in the behaviors of fibers and resins during inter-ply slipping are shown in Figure 5. During the test, the prepreg temperature can be considered to be stable. The viscosity of the resin was also constant, the *η* was stabled. In the initial stage of the test, the stretching rate of the tensile machine needs to be gradually increased to the set rate. The flow rate of the resin is changed. The change of resin layer height can be ignored. The change of parameter *v* (Equation (1)) is the main reason for the change of slip resistance. *d* = *v* × *t*, the d is the relative slipping displacement. The *t* is the time of the test. Then the slip kinetic equation of the stage was obtained.

(4) $$\tau_{1}=q_{1}\cdot \;d$$

In the stage II, the speed of the puller tended to be stable. The effect of the gradual decrease of the height of the resin layer began to be obvious. The *η* and *v* remain unchanged. The inter-ply slipping resistance begins to grow nonlinearly. It is assumed that the decrease rate of resin layer height is proportional to the increase of relative slipping displacement. Then Equation (5) was obtained.

(5) $$\tau_{2}=\frac{q_{2}}{d}+\tau_{1}^{\infty }$$

The Coulomb friction law is applied in the stage III. In the stage III, the friction interface is filled with fibers. With the increase of relative slipping displacement, the content of resin at the contact interface gradually decreases, the content of fiber gradually increases, and the interfacial friction coefficient gradually increases and tends to the interfacial friction coefficient of pure fiber. The change of inter-ply slipping resistance was mainly due to the change of interfacial friction coefficient with the change of slip distance. Then Equation (6) was obtained.

(6) $$\tau_{3}=q_{3}\cdot \;d+\tau_{2}^{\infty }$$

where the $\tau_{1}$, $\tau_{2}$, $\tau_{3}$ are slip stress (MPa) at different stage. The d is relative slipping displacement (mm). $\tau_{1}^{\infty }$ is the stress of yield point. and $\tau_{2}^{\infty }$ is the stress of hardening point. $q_{1}$, $q_{2}$ and $q_{3}$ are parameters of models. The $q_{1}$ and $q_{2}$ are related to the viscosity of the resin (*η*) and relative slip rate (*v*). The $q_{3}$ is related to the interface friction coefficient and interface pressure. 

**Figure 6:**
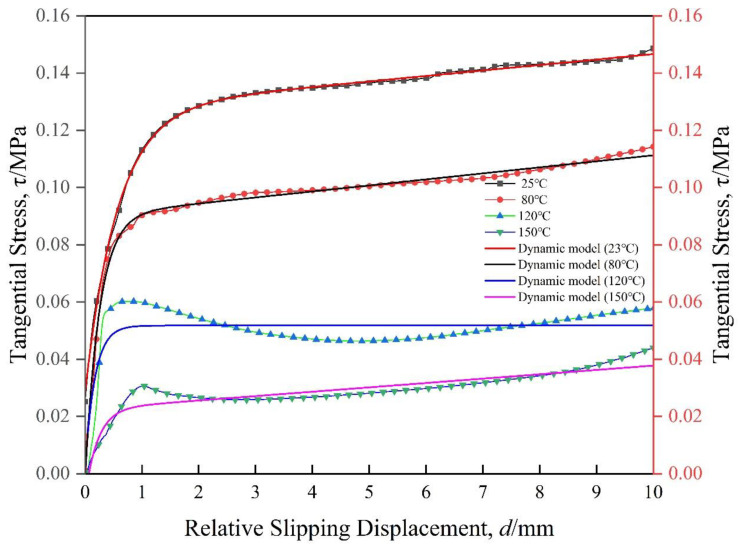
The comparison between the test results and the dynamic model test results.

**Figure 7:**
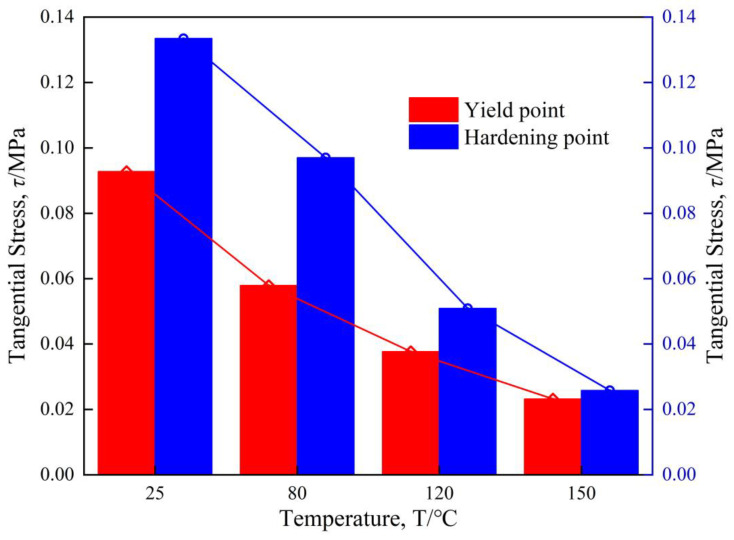
Tangential stress of transformation point.

The inter-ply slipping kinetic model parameters of composite prepregs are shown in Table 1. The comparison between the test results at different temperatures and the dynamic model test results is shown in the Figure 6. The Figure 7 shows the critical tangential stress of each stage at different temperatures. The results show that the critical tangential stress decreases with the increase of temperature. This is because the viscosity of the prepreg decreases as the temperature increases. Therefore, at a temperature where the viscosity of the prepreg is low, the preforming manufacturing will be carried out, and the slip will be smooth, which is beneficial to reduce the generation of wrinkles.

## 6. Verified by Finite Element Simulation

### 6.1. Finite Element Model and Boundary Conditions

**Figure 8:**
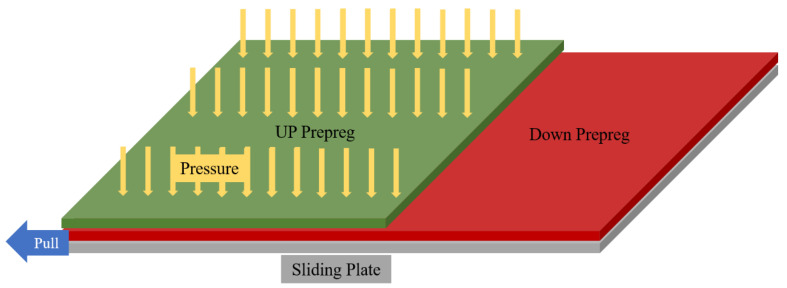
Diagram of 1/2 finite element model.

The inter-ply slipping test has symmetry. The 1/2 finite element model of inter-ply slipping test was established (Figure 8). The prepregs was defined by ‘shell’ element. The homogenization model was used to characterize the properties of prepregs. The type of material is ‘Shell, Homogeneous’. The modulus and other properties of the prepregs were input by ‘Engineering Constants’. In many works [26,27,28], homogeneous shell elements are used to define the properties of unidirectional carbon fiber reinforced resin composite prepregs. The sliding plate was defined by ‘Analytical rigid’. The ‘up prepreg’ refers to the prepreg attached to the pressure plate. The ‘down prepreg’ is the prepreg attached to the sliding plate. The ‘Tie’ was used to connect the down prepreg and the sliding plate. The displacement of the edge of the prepreg is limited. The pressure was applied to the surface of the up prepreg. The functions of the pressure plate and cylinder were defined. The sliding plate was given displacement. The relative slip was occurred between the up prepreg and down prepreg. The inter-ply slipping behaviors of prepregs was defined in ‘Tangential Behavior’. The inter-ply slipping behaviors of the prepreg was compiled by the ‘vfriction’ subroutine and input into the main program.

### 6.2. Verification Results

**Figure 9:**
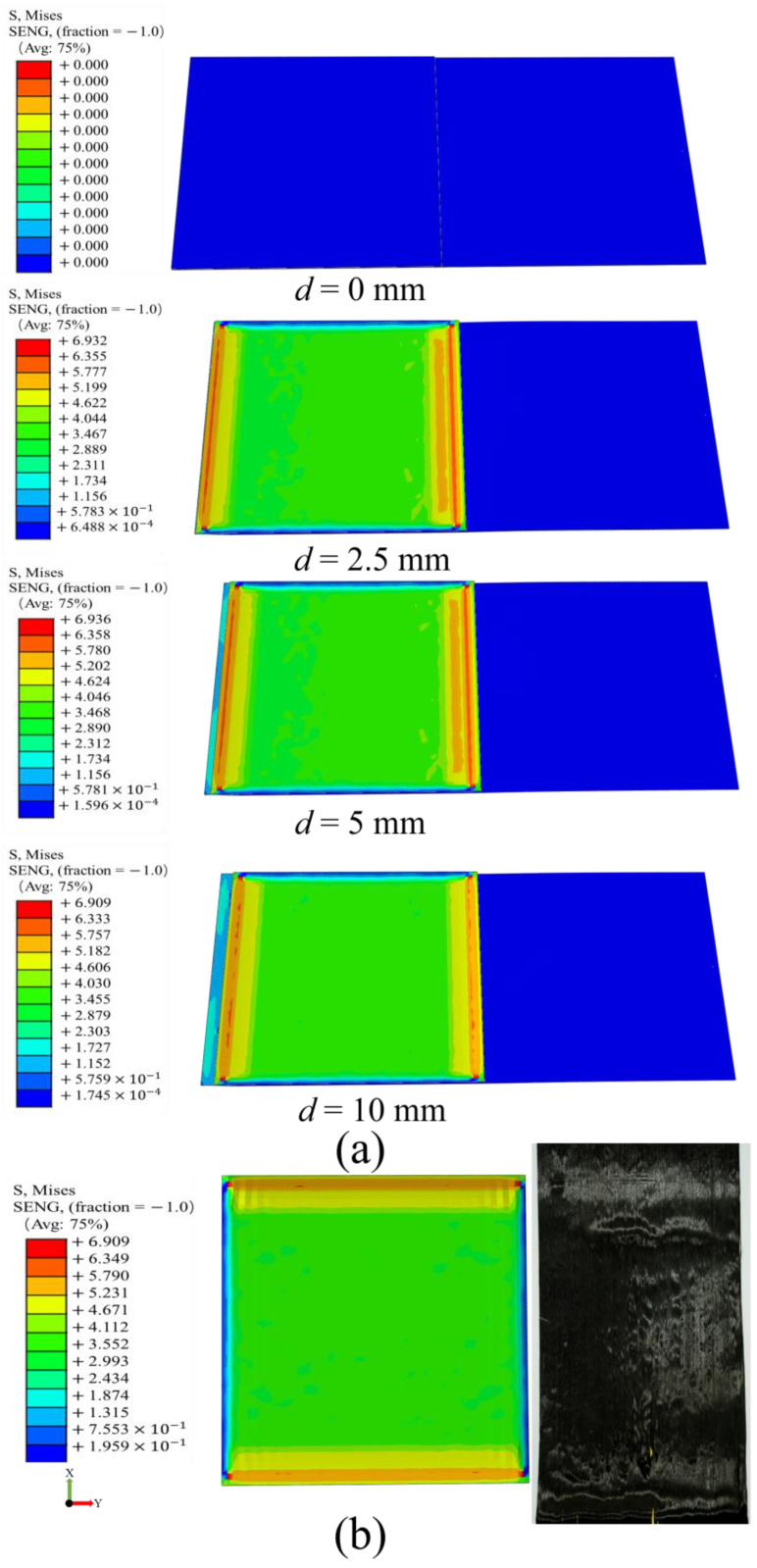
Finite element simulation result: (**a**) Process of slip behaviors simulation; (**b**) The comparison between finite element simulation and test.

**Figure 10:**
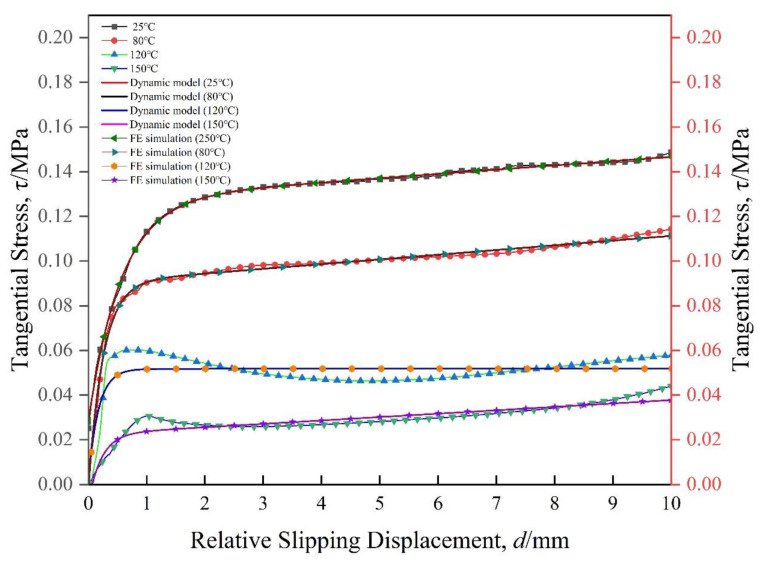
Comparison of experimental and analytical results.

Figure 9a shows the process of finite element simulation of inter-ply slipping. The stress concentration was observed at the front and end of the slip. The prepregs was continuously bonded and detached in this area. The comparison of the finite element simulation result with the slip dynamic model and the test was shown in Figure 10. The unity of the three proves the validity and correctness of the slip dynamic model. The correctness of the finite element simulation was also proved. The slip behaviors between the prepreg layers is also proved to include Coulomb friction and fluid dynamics. The surface of prepreg after slip test was observed. The surface of the prepreg become rough after the test (Figure 9b). The area of boned and detached were also observed. The correctness of the finite element simulation results was further proved.

## 7. Finite Element Simulation

**Figure 11:**
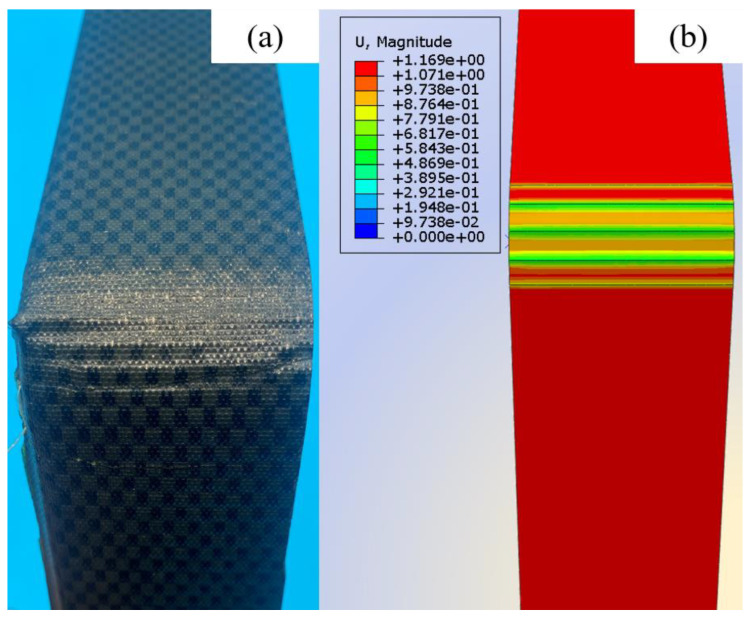
The C-type part through the hot diaphragm preforming process and the simulation result are compared: (**a**) C-type part; (**b**) the simulation result.

The Figure 11 shows the comparison between the actual wrinkle and the simulated wrinkle. It can be seen from the comparison that the use of the inter-ply slipping kinetic equation is beneficial to improve the accuracy of the prediction of the position and shape of the wrinkle in the hot diaphragm preforming process.

## 8. Conclusions

In the process of hot diaphragm preforming, the inter-ply slipping resistance is one of the causes of wrinkles. Based on the vertical inter-ply slipping test system, the inter-ply slipping behaviors of carbon fiber reinforced epoxy resin prepregs were characterized. The inter-ply slipping behaviors of prepreg is divided into three stages. In the stage I, the increase of slip resistance is mainly due to the change of the relative slip rate of the interface. In the stage II, the decrease of the thickness of the resin layer leads to the change of the slip resistance, and the slip resistance caused by the contact of the fiber surface also begins to join in this stage. In the stage III, the direct contact of the fibers is the main reason for the slip resistance. The temperature will affect the viscosity of the prepreg. As the viscosity decreases, the inter-ply slipping stress of the prepreg will also decrease. The critical slip stress of each stage also decreases with the decrease of viscosity. Controlling the molding temperature during the preforming of the hot diaphragm to keep the viscosity of the prepreg at a low level can reduce the slip stress and reduce the generation of wrinkles. Based on the Coulomb friction law and the fluid dynamics equation, the inter-ply slipping dynamics equation is established. The three stages of inter-ply slipping behaviors of prepreg are characterized. It is expressed in ABAQUS through the ‘vfriction‘ subroutine. The inter-ply slipping dynamic equation is introduced into the finite element simulation work, and the prediction accuracy of the shape and position of the fold is improved.

## Figures and Tables

**Table 1:** Inter-ply slipping kinetic model parameters of composite prepregs.

Temperature (°C)	q_1_	q_2_	q_3_	$\tau_{1}^{\infty }$	$\tau_{2}^{\infty }$	R^2^
25	0.14696	−0.03076	0.12702	0.09278	0.13349	0.99872
80	0.20291	−0.01672	0.00211	0.0579	0.097	0.99110
120	0.253	−0.01273	0.00082	0.0377	0.0509	0.95355
150	0.20697	−0.00604	0.00197	0.02318	0.02581	0.96907

## Data Availability

The original contributions presented in the study are included in the article, further inquiries can be directed to the corresponding author.

## References

[1] Erland S., Dodwell T.J., Butler R. (2015). Characterisation of inter-ply shear in uncured carbon fibre prepreg. Compos. Part A Appl. Sci. Manuf..

[2] Fetfatsidis K.A., Gamache L.M., Gorczyca J.L., Sherwood J.A., Jauffrès D., Chen J. (2013). Design of an apparatus for measuring tool/fabric and fabric/fabric friction of woven-fabric composites during the thermostamping process. Int. J. Mater. Form..

[3] Fetfatsidis K.A., Jauffrès D., Sherwood J.A., Chen J. (2013). Characterization of the tool/fabric and fabric/fabric friction for woven-fabric composites during the thermostamping process. Int. J. Mater. Form..

[4] Kaushik V., Raghavan J. (2010). Experimental study of tool–part interaction during autoclave processing of termoset polymer composite structures. Compos. Part A Appl. Sci. Manuf..

[5] (2024). Jiani Yu, Lidong Wang, Bin Shao, Yingying Zong, The role of graphene inter-ply slippingping on the deformation behavior of graphene/copper composite. J. Alloys Compd..

[6] Hozjan T., Saje M., Srpčič S., Planinc I. (2013). Geometricall and materially non-linear analysis of planar composite structures with an inter-ply slipping. Comput. Struct..

[7] Yu X., Zhang L., Mai Y. (2003). Modelling and finite element treatment of intra-ply shearing of woven fabric. J. Mater. Process. Technol..

[8] Mallon P.J., O’Bradaigh C.M., Pipes R.B. (1989). Polymeric diaphragm forming of complex-curvature thermoplastic composite parts. Composites.

[9] O’Bradaigh C.M., Mallon P.J. (1989). Effect of forming temp.erature on the properties of polymeric diaphragm formed thermoplastic composites. Compos. Sci. Technol..

[10] Zhang P., Qiao Z.-A., Zhang Z., Wan S., Dai S. (2014). Mesoporous graphene-like carbon sheet: High-power supercapacitor and outstanding catalyst support. J. Mater. Chem. A.

[11] Larberg Y.R., Åkermo M. (2011). On the interply friction of different generations of carbon/epoxy prepreg systems. Compos. Part A Appl. Sci. Manuf..

[12] Hamila N., Boisse P. (2008). Simulations of textile composite reinforcement draping using a new semi-discrete three node finite element. Compos. B Eng..

[13] Lomov S.V., Boisse P., Deluycker E., Morestin F., Vanclooster K., Vandepitte D., Verpoest I., Willems A. (2008). Full-field strain measurements in textile deformability studies. Compos. Part A Appl. Sci. Manuf..

[14] Lebrun G., Bureau M.N., Denault J. (2003). Evaluation of bias-extension and pictureframe test methods for the measurement of intraply shear properties of PP/glass commingled fabrics. Compos. Struct..

[15] Guzman-Maldonado E., Hamila N., Naouar N., Moulin G., Boisse P. (2016). Simulation of thermoplastic prepreg thermoforming based on a visco-hyperelastic model and a thermal homogenization. Mater. Des..

[16] Beakou A., Cano M., Le Cam J.B., Verney V. (2011). Modelling slit tape buckling during automated prepreg manufacturing: A local approach. Compos. Struct..

[17] Matveev M.Y., Schubel P.J., Long A.C., Jones I.A. (2016). Understanding the buckling behaviour of steered tows in Automated Dry Fibre Placement (ADFP). Compos. Part A Appl. Sci. Manuf..

[18] Belhaj M., Hojjati M. (2018). Wrinkle formation during steering in automated fiber placement: Modeling and experimental verification. Reinf. Plast. Compos..

[19] Hallander P., Sjölander J., Petersson M., Åkermo M. (2016). Interface manipulation towards wrinkle-free forming of stacked UD prepreg layers. Compos. Part A Appl. Sci. Manuf..

[20] Bakhshi N., Hojjati M. (2018). An experimental and simulative study on the defects appeared during tow steering in automated fiber placement. Compos. Part A Appl. Sci. Manuf..

[21] Dörr D., Henning F., Kärger L. (2018). Nonlinear hyperviscoelastic modelling of intra-ply deformation behaviour in finite element forming simulation of continuously fibre-reinforced thermoplastics. Compos. Part A Appl. Sci. Manuf..

[22] Zhao Y., Zhang T., Li H., Zhang B. (2020). Characterization of prepreg-prepreg and prepreg-tool friction for unidirectional carbon fiber/epoxy prepreg during hot diaphragm forming process. Polym. Test..

[23] Wang L., Xu P., Peng X., Zhao K., Wei R. (2019). Characterization of inter-ply slipping behaviors in hot diaphragm preforming: Experiments and modelling. Compos. Appl. Part A Sci. Manuf..

[24] Grewal H.S., Hojjati M. (2017). Inter-ply Friction of Unidirectional Tape and Woven Fabric Out-of-autoclave Prepregs. Int. J. Compos. Mater..

[25] Thije R.H.W.T., Akkerman R., Ubbink M., van der Meer L. (2011). A lubrication approach to friction in thermoplastic composites forming processes. Compos. Appl. Sci. Manuf..

[26] Larberg Y., Åkermo M. (2014). In-plane deformation of multi-layered unidirectional thermoset prepreg–Modelling and experimental verification. Compos. Appl. Sci. Manuf..

[27] Li Z., Liu W., Sun B., Yue G., Tan Y., Zhang J. (2022). A numerical approach to characterize the compression and relaxation behavior of uncured prepreg laminates in the process of hot press-forming. Mater. Res. Express.

[28] Li Z., Liu J., Liu W., Liu W., Song Q., Chen P., Chen P.-H., Yue G. (2024). Determination of lubricating behavior of resin during the sliding process of unidirectional prepreg. Mater. Res. Express.

